# Acute Liver Failure Associated With Intravenous Amiodarone: A Case Report

**DOI:** 10.7759/cureus.107853

**Published:** 2026-04-28

**Authors:** Nikolaos I Davanellos, Rafail Giannas, Nikolaos I Kouris, Dimitrios Argiropoulos, Michalis Apergis

**Affiliations:** 1 Department of Internal Medicine, General Hospital of Syros "Vardakeio and Proio", Ermoupoli, GRC

**Keywords:** acute liver failure, amiodarone, atrial fibrillation, drug-induced liver injury, fulminant hepatic failure, hepatotoxicity, intravenous amiodarone

## Abstract

Amiodarone is a widely used class III antiarrhythmic agent that is indicated for the management of supraventricular and ventricular arrhythmias. Although mild elevations in liver enzymes are relatively common during therapy, severe hepatotoxicity and acute liver failure remain rare, yet potentially fatal complications, particularly when following intravenous administration. We report the case of an 80-year-old male who presented with palpitations and was diagnosed with atrial fibrillation with rapid ventricular response. Intravenous amiodarone was initiated for rhythm control. Within 14 hours, the patient developed rapidly progressive hepatocellular injury with marked elevation of transaminases, hyperbilirubinemia, and severe coagulopathy. Liver injury occurred while the patient remained hemodynamically stable, making ischemic hepatitis less likely, although it could not be completely excluded. Viral hepatitis was excluded, and imaging demonstrated no biliary obstruction. The patient’s condition deteriorated rapidly, progressing to fulminant hepatic failure, metabolic acidosis, and multiorgan dysfunction, ultimately resulting in death despite aggressive supportive management.

This case highlights a possible association between intravenous amiodarone and acute liver failure. While the temporal relationship suggests a drug-induced liver injury, a multifactorial etiology, including potential contributions from hypoxia or other systemic factors, cannot be entirely excluded. This report underscores the importance of early recognition and careful monitoring for severe hepatic complications in patients receiving intravenous amiodarone.

## Introduction

Amiodarone is a commonly used class III antiarrhythmic agent that is indicated for the treatment of both supraventricular and ventricular arrhythmias. It is widely used in the management of acute ventricular tachycardia and ventricular fibrillation following unsuccessful defibrillation, as well as for the long-term treatment of refractory supraventricular arrhythmias such as atrial fibrillation [1]. Owing to its efficacy in controlling arrhythmias, amiodarone is frequently used in both acute and chronic clinical settings [2].

Notwithstanding its therapeutic benefits, amiodarone is linked to several adverse effects involving multiple organ systems, including the thyroid, lungs, skin, and liver [2,3]. Hepatotoxicity associated with amiodarone therapy is well documented. Mild and asymptomatic elevations in liver transaminases occur in up to 15%-50% of patients receiving the drug and are usually reversible after reducing the dose or discontinuing therapy [4,5]. Current recommendations suggest monitoring liver function tests during treatment and discontinuation of the drug if transaminase levels exceed two to three times the upper limit of normal [6]. Severe hepatotoxicity associated with intravenous amiodarone remains rare but clinically significant, with reported cases describing rapid progression of hepatocellular injury, often leading to acute liver failure shortly after drug initiation [7,8].

Importantly, intravenous amiodarone differs from oral formulations due to the presence of polysorbate 80, a solvent that has been proposed as a contributing factor to hypotension, impaired hepatic perfusion, and potential mitochondrial dysfunction. This distinction may partly explain the more abrupt and severe presentation of hepatocellular injury observed in intravenous cases.

In particular, intravenous amiodarone has been associated with cases of rapidly progressive liver injury characterized by dramatic elevations in transaminases and, in rare instances, acute liver failure [8]. Early recognition of this complication is crucial, as prompt discontinuation of the drug may prevent further deterioration. Here, we present a case of rapidly progressive acute liver failure following intravenous amiodarone administration, highlighting the importance of early recognition of this rare but potentially fatal complication.

## Case presentation

Initial presentation

An 80-year-old male presented to the emergency department with palpitations, tachycardia, and generalized malaise. His past medical history was significant for atrial fibrillation, a prior ischemic stroke, and previous gastrointestinal bleeding. He was an active smoker. His regular medications included olmesartan, rivaroxaban, bisoprolol, and oral iron supplementation. There was no known history of chronic liver disease. Available baseline laboratory tests, including bilirubin and renal function, were within normal limits. Serum albumin levels were not available.

On presentation, the patient was alert and oriented with a Glasgow Coma Scale (GCS) score of 15/15. Vital signs demonstrated blood pressure of 172/85 mmHg, heart rate of 150 beats/min, oxygen saturation of 90% on room air, and body temperature of 36°C. Physical examination revealed an irregular tachyarrhythmia consistent with atrial fibrillation with rapid ventricular response. Pulmonary auscultation revealed crackles at the right lung base along with scattered rhonchi. The abdomen was soft and non-tender.

**Figure 1:**
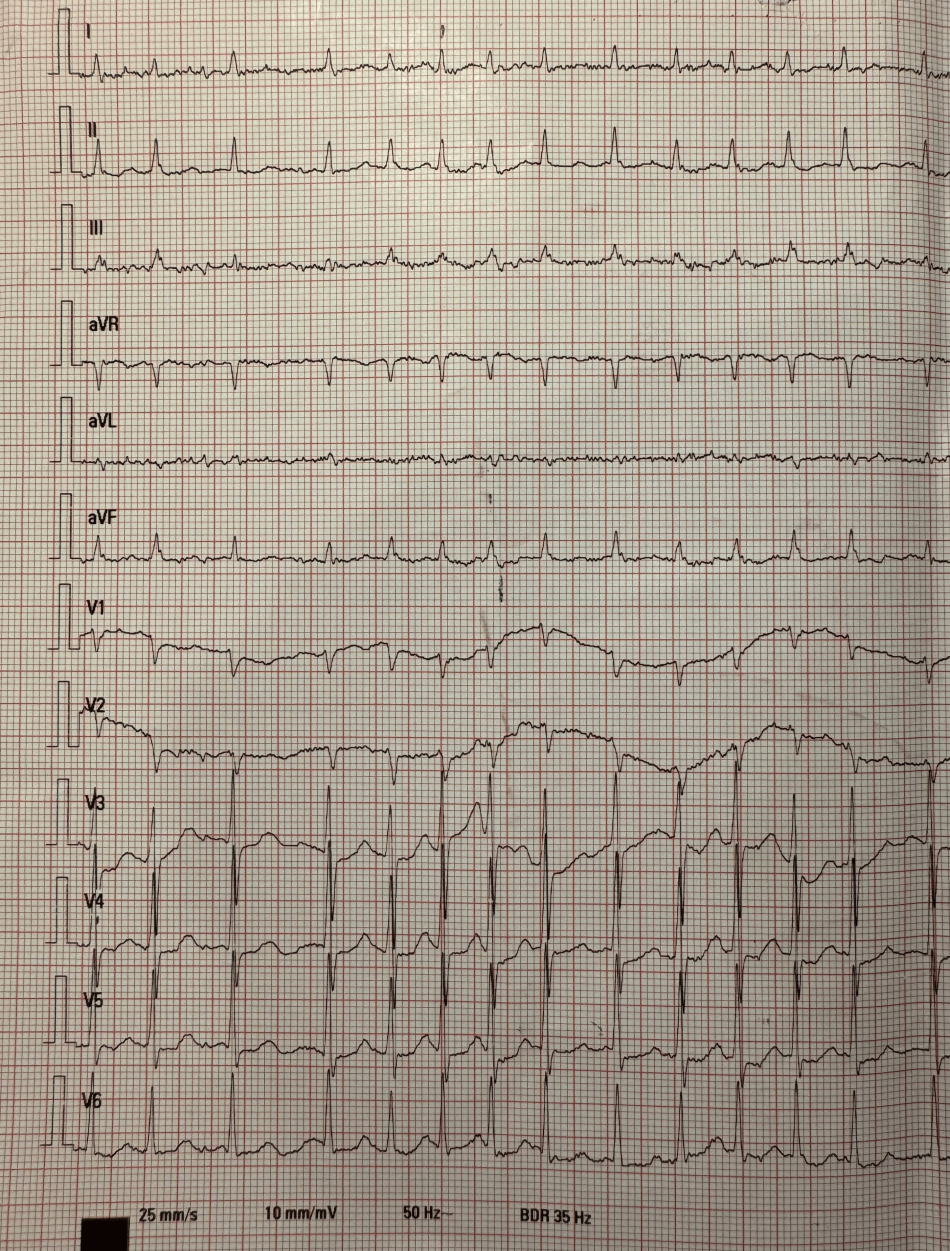
Electrocardiogram showing atrial fibrillation with rapid ventricular response

Electrocardiography confirmed atrial fibrillation with rapid ventricular response (Figure 1). The patient was admitted for rate and rhythm control. Intravenous amiodarone infusion was initiated. The treatment regimen consisted of two ampoules of amiodarone (150 mg/3 mL each) diluted in 250 mL of 5% dextrose solution administered initially at approximately 125 mL/hour, followed by eight ampoules diluted in 500 mL of 5% dextrose administered at 40 mL/hour. His beta-blocker therapy (bisoprolol) was also titrated. The relatively high cumulative dose and rapid infusion rate may have contributed to the severity of toxicity.

In the first hours after his admission, the patient developed progressive dyspnea and diaphoresis. Oxygen therapy was given using a 100% non-rebreather mask, and intravenous methylprednisolone (125 mg) was administered. Rapid antigen testing for COVID-19 and influenza was negative.

Imaging findings

Computed tomography imaging was performed to further evaluate the patient's respiratory deterioration. A non-contrast CT scan of the brain showed no evidence of intracranial hemorrhage or acute ischemic pathology. Chronic gliotic changes consistent with a prior ischemic stroke were observed.

**Figure 2:**
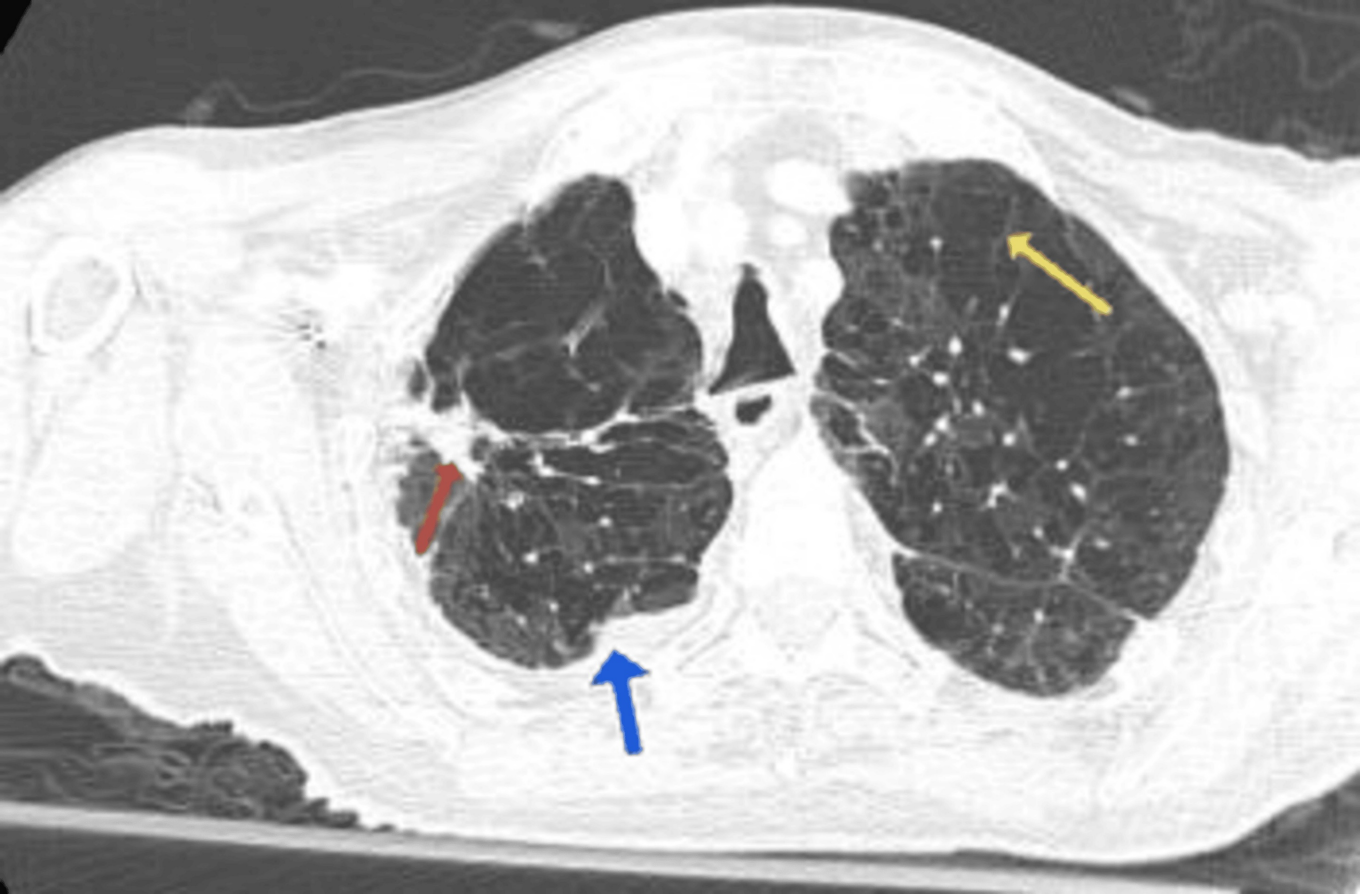
Chest CT demonstrating extensive emphysematous changes (yellow arrow), a right upper lobe spiculated lesion suspicious for malignancy (red arrow), and a right-sided pleural effusion (blue arrow)

Non-contrast CT imaging of the chest revealed extensive bilateral emphysematous changes. An irregular lesion with spiculated margins was identified in the anterior segment of the right upper lobe extending toward the pulmonary hilum, raising suspicion for a possible malignant pulmonary lesion. Multiple cystic changes were noted in the middle lobe and bilateral lower lobes. Right-sided pleural thickening with calcifications was present along with a small bilateral pleural effusion, more prominent on the right side, accompanied by adjacent compressive atelectasis. Mild mediastinal lymphadenopathy was also identified (Figure 2).

Contrast-enhanced CT imaging of the thorax was subsequently performed to exclude pulmonary embolism. No filling defects were observed in the main or segmental pulmonary arteries.

**Figure 3:**
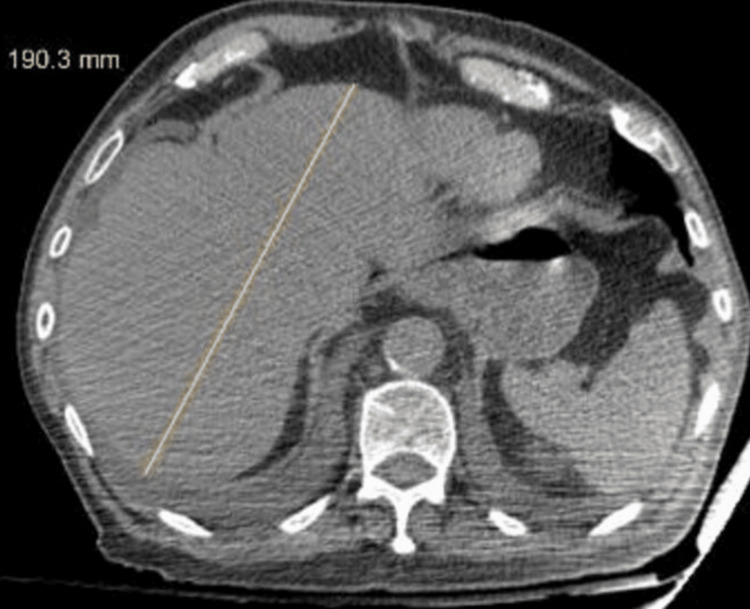
Abdominal CT demonstrating hepatomegaly (liver span approximately 19 cm) without focal hepatic lesions

Contrast-enhanced CT imaging of the abdomen demonstrated mild hepatomegaly without focal hepatic lesions. There was no detection of dilation of the intrahepatic or extrahepatic biliary ducts. The gallbladder appeared contracted. The pancreas, spleen, kidneys, and adrenal glands showed no focal abnormalities. Multiple bilateral cortical renal cysts were observed. The urinary bladder contained intraluminal air with a diverticulum in the right lateral wall. The prostate gland appeared enlarged with smooth contours. No intra-abdominal free fluid was detected (Figure 3).

Clinical course

Owing to concern for possible infection and respiratory deterioration, intravenous antibiotic therapy with piperacillin-tazobactam was initiated. Oxygen therapy was escalated to high-flow nasal oxygen. At that stage, the patient remained hemodynamically stable, with blood pressure measurements approximately 135-140/75-85 mmHg and preserved urine output.

Within approximately 14 hours after initiation of intravenous amiodarone, laboratory testing demonstrated rapidly progressive hepatocellular injury. Liver enzymes were markedly elevated with aspartate aminotransferase (AST) 2647 U/L and alanine aminotransferase (ALT) 1708 U/L, accompanied by lactate dehydrogenase (LDH) 4487 U/L, hyperbilirubinemia, and severe coagulopathy with an international normalized ratio (INR) 6.4 (Table 1). It has to be noted that these abnormalities occurred while the patient remained hemodynamically stable without preceding hypotension, making primary ischemic hepatitis less likely, although not fully excluding a contributory hypoxic component. Viral hepatitis testing, including hepatitis A, B, and C serology, was negative. Although the patient was receiving rivaroxaban, we acknowledge that direct oral anticoagulants may variably affect INR values and do not reliably reflect anticoagulant activity. Therefore, INR values were interpreted with caution and not attributed solely to anticoagulation therapy. The temporal sequence of events (intravenous amiodarone initiation → rapid rise in liver enzymes → subsequent hemodynamic deterioration) supports a drug-induced pattern of liver injury.

**Table 1:** Evolution of liver function tests and coagulation parameters following intravenous amiodarone administration * Elevated at baseline; the patient was receiving rivaroxaban, and INR values may not reliably reflect anticoagulant activity. AST: aspartate aminotransferase; ALT: alanine aminotransferase; LDH: lactate dehydrogenase; INR: international normalized ratio

Parameter	Reference range	At admission	14 hours after amiodarone	Peak value
AST (U/L)	10-45	22	2647	7581
ALT (U/L)	7-45	15	1708	4240
LDH (U/L)	0-248	207	4487	7912
Total bilirubin (mg/dL)	0-1.2	1.32	5.62	5.24
INR	0.8-1.2	3.5*	6.4	8.7
Creatinine (mg/dL)	0.81-1.44	0.94	1.33	2.01
Lactate (mmol/L)	0.5-2.2	-	-	>15

Notwithstanding initial hemodynamic stability, the patient’s clinical condition subsequently deteriorated. He developed severe hypotension with blood pressure decreasing to 70/60 mmHg and a reduced level of consciousness with GCS 10/15 (E4, M5, V1) and progressive respiratory failure.

Arterial blood gas analysis revealed profound metabolic acidosis with pH <6.80, pCO_2_ 71 mmHg, pO_2_ 59 mmHg, and lactate >15 mmol/L (Table 2), indicating severe tissue hypoperfusion. Inflammatory markers, including C-reactive protein (CRP) and procalcitonin, were not markedly elevated. Although empirical antibiotic therapy was initiated due to suspected infection and respiratory deterioration, there was no definitive microbiological evidence of sepsis at the time of liver injury onset.

**Table 2:** Arterial blood gas analysis before intubation

Parameter	Value	Reference range
pH	<6.80	7.35-7.45
pCO_2_	71 mmHg	35-45
pO_2_	59 mmHg	80-100
Lactate	>15 mmol/L	0.5-2.2

Vasopressor support was initiated with norepinephrine infusion (four ampules diluted in 250 mL normal saline), resulting in a transient improvement of blood pressure to approximately 140/70 mmHg.

The patient received intravenous sodium bicarbonate (four ampoules in total) for severe metabolic acidosis. Coagulopathy was treated with vitamin K administration and transfusion of two units of fresh frozen plasma, while anticoagulation therapy with rivaroxaban was discontinued. A continuous infusion of furosemide (25 ampules) was initiated because of worsening renal function and oliguria.

Given progressive respiratory failure and severe metabolic derangement, the patient required endotracheal intubation, performed using cisatracurium and midazolam. A bedside transthoracic echocardiogram performed after intubation demonstrated a mildly reduced left ventricular ejection fraction (~45%) without evidence of acute cardiac dysfunction, making primary cardiogenic shock unlikely.

The presence of respiratory deterioration, systemic instability, and elevated lactate raised the possibility of a multifactorial process, including sepsis-related liver injury and hypoxic hepatitis. The imaging findings of a spiculated lung lesion raised suspicion for an underlying malignancy, which may have contributed to the patient’s overall clinical vulnerability and systemic condition. In addition, the presence of significant respiratory pathology and subsequent respiratory failure may have contributed to hepatic hypoxia, representing a potential confounding factor in the development of liver injury.

While the early onset of marked hepatocellular injury shortly after intravenous amiodarone administration supports a primary drug-induced mechanism, the subsequent development of hypotension and respiratory failure suggests that secondary hypoxic or ischemic factors may have contributed to the progression and severity of liver injury. The markedly elevated LDH levels further support the possibility of a hypoxic or ischemic component, as such elevations are commonly observed in ischemic hepatitis. Further laboratory testing demonstrated massive hepatocellular injury with peak AST 7581 U/L and ALT 4240 U/L, accompanied by severe coagulopathy (INR 8.7) and acute kidney injury, consistent with fulminant hepatic failure and multiorgan dysfunction (Table 1). Despite aggressive supportive management including mechanical ventilation, vasopressor support, correction of metabolic acidosis, broad-spectrum antimicrobial therapy, and transfusion support, the patient remained hemodynamically unstable.

The clinical course rapidly progressed to refractory shock and multiorgan failure, and the patient ultimately succumbed to fulminant hepatic failure. The temporal association with intravenous amiodarone administration, early onset of hepatocellular injury, and exclusion of alternative etiologies support a diagnosis of acute amiodarone-induced hepatotoxicity, likely with a multifactorial contribution including hypoxic injury. Although ischemic hepatitis cannot be completely excluded, the absence of hypotension during the early phase of liver injury and the close temporal relationship with intravenous amiodarone administration make a primary drug-induced mechanism more likely.

## Discussion

Amiodarone is a widely used antiarrhythmic agent with complex pharmacologic properties and a broad range of clinical applications. Although its effectiveness in the management of both supraventricular and ventricular arrhythmias is well established, the drug is also associated with multiple organ toxicities, including pulmonary, thyroid, neurologic, dermatologic, and hepatic complications [2,3,9].

Hepatic toxicity associated with amiodarone therapy most commonly manifests as mild, transient elevations in liver enzymes. The specific abnormalities are frequently asymptomatic and are usually reversible after dose reduction or discontinuation of therapy [4,5]. Nevertheless, more severe hepatic injury has been described, including acute hepatitis, cholestatic injury, cirrhosis, and fulminant hepatic failure [6,7].

Acute liver injury following intravenous amiodarone administration is a rare but well-documented complication. Several reports have described rapidly progressive hepatocellular injury occurring within hours to days after initiation of intravenous amiodarone therapy, often characterized by dramatic elevations in serum transaminases and, in severe cases, progression to acute liver failure [8,10].

The exact mechanism underlying this form of hepatotoxicity remains incompletely understood. One proposed mechanism involves direct hepatocellular toxicity mediated by polysorbate 80, the solvent used in the intravenous formulation of amiodarone [11]. Experimental observations have suggested that polysorbate 80 may contribute to hypotension and hepatocellular injury, potentially through mitochondrial dysfunction and impaired hepatic perfusion [10,11]. Another proposed mechanism involves ischemic hepatitis secondary to drug-induced hypotension or circulatory instability [12].

In the present case, however, marked elevations in liver enzymes occurred approximately 14 hours after initiation of intravenous amiodarone while the patient remained hemodynamically stable, with preserved blood pressure and no preceding hypotension. This temporal relationship, in the setting of preserved hemodynamic stability and absence of hypotension during the early phase of liver injury, makes primary ischemic hepatitis less likely. However, this does not fully exclude a contributory ischemic or hypoxic component.

Notably, subsequent clinical deterioration, including hypotension and respiratory failure, raises the possibility that liver injury may be multifactorial, with both drug-induced and ischemic mechanisms contributing. The markedly elevated LDH levels observed in this case are typically associated with ischemic hepatitis, further supporting a possible hypoxic or ischemic contribution to the observed liver injury.

Serum lactate levels were not measured during the early phase of liver injury; however, a marked elevation was observed later in the clinical course (>15 mmol/L), supporting the presence of secondary hypoperfusion and tissue hypoxia rather than a primary ischemic trigger.

Alternative causes of acute liver injury were systematically excluded. Viral hepatitis testing was negative, and imaging studies did not demonstrate biliary obstruction or structural hepatic disease. The rapid onset of hepatocellular injury following drug administration, together with the absence of alternative etiologies, is consistent with amiodarone-associated hepatotoxicity.

Although the temporal association suggests a relationship with intravenous amiodarone, contributory factors such as hypoxia, systemic illness, and hemodynamic fluctuations cannot be entirely excluded. A retrospective causality assessment based on the Roussel Uclaf Causality Assessment Method (RUCAM) criteria suggests a probable drug-induced liver injury [13].

The severity of liver injury in this case may be attributed to a combination of factors. Advanced age, underlying comorbidities, and the patient’s overall clinical condition may have increased susceptibility to hepatotoxicity. In addition, the rapid intravenous administration and cumulative dose of amiodarone may have contributed to the severity of hepatic injury.

Furthermore, the presence of hemodynamic instability and hypoxia likely played a role in exacerbating liver damage, supporting a multifactorial pathophysiological process. This interpretation is further supported by causality assessment using RUCAM criteria, suggesting a probable drug-induced liver injury, although a multifactorial contribution cannot be excluded [13].

Similar cases reported in the literature describe a comparable clinical course, characterized by abrupt elevation of liver enzymes, severe coagulopathy, and rapid progression to fulminant hepatic failure shortly after intravenous amiodarone exposure [8,10,14]. Even though some patients recover after discontinuation of the drug, severe cases may progress to multiorgan failure and death despite aggressive supportive treatment [3,14].

Although rare, this complication should be considered in patients who develop abrupt elevations in transaminases shortly after initiation of intravenous amiodarone therapy. This case underscores the importance of early recognition of drug-induced liver injury in patients receiving intravenous amiodarone. Clinicians should closely monitor liver function tests following initiation of therapy and maintain a high index of suspicion for hepatotoxicity in patients who develop unexplained elevations in transaminases. Prompt discontinuation of the drug may be critical in preventing progression to fulminant hepatic failure and improving clinical outcomes.

## Conclusions

Intravenous amiodarone, although widely used, has been associated with rare cases of fulminant hepatic failure with a rapid and catastrophic course. Early recognition of hepatotoxicity and prompt discontinuation of the drug may be critical in preventing progression to severe liver injury. Clinicians should remain vigilant for sudden elevations in liver enzymes following initiation of therapy.

In the present case, while the temporal association suggests drug-induced liver injury related to intravenous amiodarone, a multifactorial etiology, with potential contributions from hypoxia, systemic illness, and hemodynamic instability, cannot be excluded.
